# Self-perceived physical health predicts cardiovascular disease incidence and death among postmenopausal women

**DOI:** 10.1186/1471-2458-13-468

**Published:** 2013-05-14

**Authors:** Nazmus Saquib, Robert Brunner, Jessica Kubo, Hilary Tindle, Candyce Kroenke, Manisha Desai, Martha L Daviglus, Norrina Allen, Lisa W Martin, Jennifer Robinson, Marcia L Stefanick

**Affiliations:** 1 Stanford Prevention Research Center, Department of Medicine, Stanford University, Stanford, CA, USA; 2 University of Nevada, Reno, NE, USA; 3 Quantitative Sciences Unit, General Medical Disciplines, Department of Medicine, Stanford University, Stanford, CA, USA; 4 School of Medicine and Graduate School of Public Health, University of Pittsburgh, Pittsburgh, PA, USA; 5 Oakland Kaiser Permanente, Oakland, CA, USA; 6 Department of Preventive Medicine, Northwestern University Feinberg School of Medicine, Chicago, IL, USA; 7 George Washington University, Washington DC, USA; 8 University of Iowa, Iowa City, IA, USA

**Keywords:** Physical component summary, Mental component summary, Cardiovascular disease, All-cause death

## Abstract

**Background:**

Physical and Mental Component Summary (PCS, MCS, respectively) scales of SF- 36 health-related-quality-of-life have been associated with all-cause and cardiovascular disease (CVD) mortality. Their relationships with CVD incidence are unclear. This study purpose was to test whether PCS and/or MCS were associated with CVD incidence and death.

**Methods:**

Postmenopausal women (aged 50–79 years) in control groups of the Women’s Health Initiative clinical trials (n = 20,308) completed the SF-36 and standardized questionnaires at trial entry. Health outcomes, assessed semi-annually, were verified with medical records. Cox regressions assessed time to selected outcomes during the trial phase (1993–2005).

**Results:**

A total of 1075 incident CVD events, 204 CVD-specific deaths, and 1043 total deaths occurred during the trial phase. Women with low versus high baseline PCS scores had less favorable health profiles at baseline. In multivariable models adjusting for baseline confounders, participants in the lowest PCS quintile (reference = highest quintile) exhibited 1.8 (95%CI: 1.4, 2.3), 4.7 (95%CI: 2.3, 9.4), and 2.1 (95%CI: 1.7, 2.7) times greater risk of CVD incidence, CVD-specific death, and total mortality, respectively, by trial end; whereas, MCS was not significantly associated with CVD incidence or death.

**Conclusion:**

Physical health, assessed by self-report of physical functioning, is a strong predictor of CVD incidence and death in postmenopausal women; similar self-assessment of mental health is not. PCS should be evaluated as a screening tool to identify older women at high risk for CVD development and death.

## Background

Health-Related-Quality-of-Life (HRQOL) is strongly associated with cardiovascular disease (CVD) specific and all-cause mortality risk in older adults but its association with CVD incidence is unknown [1-7]. It is plausible that HRQOL will be associated with incident CVD since it is correlated with a set of risk factors (e.g. obesity, physical inactivity, and low-fiber diet) that are common to both CVD and mortality [8,9]. There is evidence that HRQOL is associated with specific types of CVD, specifically coronary heart disease and stroke [10,11]. Establishing relationships between HRQOL with incident CVD, the leading cause of death in the U.S., has practical implications as it could be used to screen individuals who are at risk of CVD development [12].

Of the HRQOL instruments, Short-Form (SF)-36-survey is widely used in research and is represented by its two summary scores: Physical Component Summary (PCS) and Mental Component Summary (MCS) [13]. PCS generally signifies the physical domain of health and exhibits a stronger association with mortality than MCS (i.e. mental domain of health) in the adjusted models; both of their effects on mortality are independent of their correlation with obesity, physical inactivity, and a low-fiber diet [1,2,6,14].

The Women’s Health Initiative (WHI), a large US-based study of postmenopausal women had baseline assessment of SF-36, adjudicated CVD outcomes, and long follow-up. We aimed to assess the relationship of SF-36 summary scores (i.e. PCS and MCS) with CVD outcomes and all-cause mortality. We hypothesized that women with low PCS and/or MCS scores would be at increased risk of developing CVD as well as at increased risk of dying from CVD specifically or from all causes.

## Methods

### Overview of the WHI

Details of eligibility criteria, data collection, and ascertainment of health outcomes in the WHI Study have been reported previously [15]. Briefly, the Clinical Trials (CT) included 68,132 post-menopausal women (ages 50–79 years) who were recruited at 40 clinical centers throughout the U.S. and randomly assigned to one or more clinical trials: the Dietary Modification (DM) trial, the Hormone Therapy (HT) trials, and/or the Calcium and Vitamin D (CaD) trial. Participants were ineligible if they had medical conditions with a predicted survival of 3 years or less, or had any condition that made them unable to adhere to study interventions (i.e. alcohol or drug-dependency, or dementia), or if they were enrolled in another trial. Women who were ineligible or unwilling to join either the HT or DM trials were invited to join an Observational Study (OS), which enrolled 93,676. Study procedures and protocols were approved by the Institutional Review Boards at the National Institute of Health and at all forty participating institutions; participants provided written informed consent (Additional file 1: Table S1).

### Rationale for sample selection

**Figure 1:**
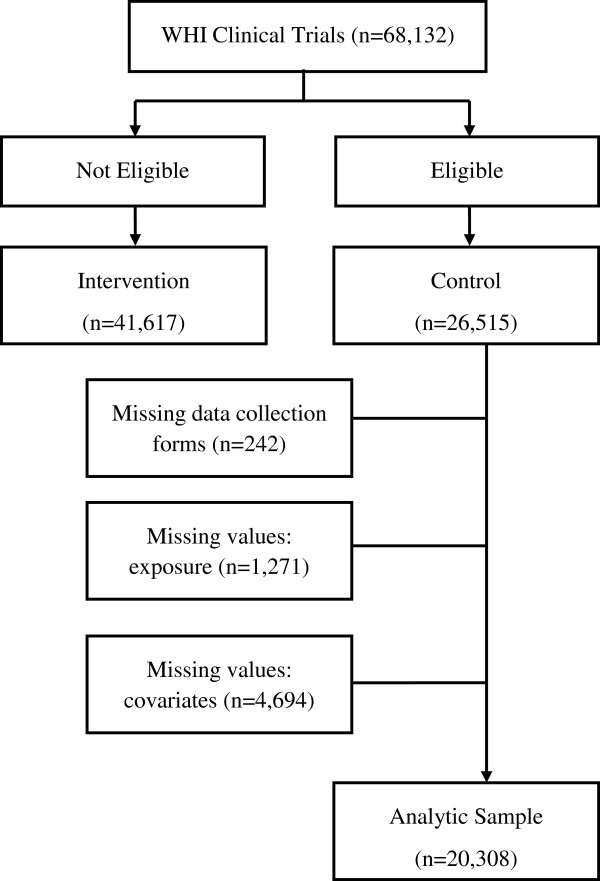
Sample selection flow chart.

These analyses were limited to the 26,515 control participants (all 3 trials) to ensure that the various trial interventions (i.e. hormone therapy, dietary modification, calcium and vitamin D) did not confound the study results. Of these, 5,965 were excluded from the analyses due to missing data at baseline: 242 participants did not fill out data collection forms, 1,271 had missing SF-36 values, and 4,694 were missing key covariate values in the statistical model. Complete data on exposure, outcomes, and specified covariates were available for the remaining 20,308 women (Figure 1).

### SF-36 quality-of-life assessments

At trial entry, participants completed the SF-36 HRQOL survey. The SF-36 assesses four physical subscales: (1) general health perceptions, (2) physical functioning (i.e. the ability to perform vigorous or moderate intensity activities that one might do in a typical day), (3) bodily pain, and (4) role limitations (i.e. problem with work and other regular daily activities due to physical health; and four mental subscales: (1) mental health, (2) vitality, (3) role limitations due to emotional problems, and (4) social functioning) [16,17]. For each subscale, overall scores were obtained by summing responses to the individual questions linked to that particular subscale. Each subscale score ranged from 0 to 100, with 0 denoting ‘bad health’ and 100 denoting ‘good health’. These scales have been shown to be reliable and have adequate construct validity in populations with a variety of disease-specific medical diagnoses [18]. All eight subscales are included in the computation of the physical and the mental component summary scores (PCS and MCS respectively), but the weight of each individual subscale varies according to algorithms specified by the SF-36 survey developers [13,16].

### Other assessments

Standardized questionnaires administered at trial entry ascertained demographic characteristics including age, ethnicity (White, African-American, Asian or Pacific Islander, Hispanic/Latino, American Indian or Alaskan Native, and Other), education level (less than high-school, high-school, some college education, and college degree), marital status (never married, married, and divorced/widowed), employed (yes, no), drinking status (none, past, current), and smoking status (current, past, and non-smokers). Pack-years of cigarette smoking were computed by multiplying the midpoint of the smoking frequency interval with the midpoint of the duration interval and dividing the product by 20 [19]. Fruits and vegetables consumption was assessed with a standardized Food Frequency Questionnaire (FFQ) and categorized as <3, 3–5, and 5+ servings/day [20].

Weight and height were measured by clinic staff at baseline with the participants wearing light clothing and no shoes. Body mass index (BMI) was calculated as weight in kilograms divided by height in meters squared and was categorized as normal (< 25 kg/m^2^), overweight (25–29.9 kg/m^2^), or obese (≥30 kg/m^2^). Systolic and diastolic blood pressures were measured according to a standardized protocol and were expressed in millimeter of mercury.

Physical activity (PA) at baseline was assessed using a validated 9-item measure of physical activity from the ‘Personal Habits Questionnaire’ and was expressed in metabolic equivalent of task (MET)-minutes per week [21]. Participants were categorized as ‘sedentary’ (<30 minutes of physical activity weekly or <90 MET/wk), ‘insufficient PA’ (90 – 449 MET/wk), ‘meets the recommended level’ (450 – 600 MET/wk), or ‘exceed the recommended level’ (> 600 MET/wk).

Health related information came from the ‘Medical History Questionnaire’ administered at baseline. Participants were asked whether they had ever received a physician’s diagnosis of: diabetes, hypertension, cardiovascular disease, asthma, emphysema, cancer except non-melanoma skin cancer, Alzheimer’s disease, multiple sclerosis, and Parkinson’s disease; or whether they were on cholesterol-lowering medication.

### Outcomes assessment

Three WHI primary outcomes were selected: (1) time to incident CVD (fatal and non-fatal), (2) time to death due to CVD, and (3) time to death from any cause. A CVD event was defined as having been diagnosed with any of the following conditions during follow-up: coronary heart disease, stroke, congestive heart failure, angina, peripheral vascular disease, carotid artery disease, and coronary revascularization. The information on the outcomes was probed semi-annually by study personnel; any report of outcome was documented and corroborated with medical records. The underlying cause of death was classified on the basis of the death certificate, medical records, and other records such as autopsy reports.

The analysis focused on the WHI trial phase (1993–2005). For incident CVD, follow-up was censored at the last follow up visit, end of trial, or date of death due to any cause, whichever occurred first. For CVD-specific death, follow-up was censored at the last follow up visit, end of trial, or date of death due to any cause other than CVD, whichever occurred first. For all-cause death, follow-up was censored at last follow-up visit or end of trial, whichever occurred first.

### Statistical analyses

Exposure and covariates were measured at baseline and the outcomes were measured at follow-up. A complete case analysis was employed. A comparison between those who were eligible and included in the analysis (N = 20,308) and those who were eligible but excluded in the analysis due to missing exposure or scientific model variables (N = 5,965) revealed several significant differences, when these two distributions were compared by Chi-square tests of association. Differences were not more than 2-3% for a given variable between the included and excluded participants. For example, 10.33% of those who were excluded died during follow up, compared to 9.06% of those who were included, with p-value 0.007.

For the ease of interpretation, PCS and MCS scores were categorized as quintiles. Correlation between PCS and MCS quintiles was evaluated with Spearman correlation statistics. Cumulative survival of the outcome events (CVD incidence, CVD death, and all-cause death) in each of the PCS and MCS quintiles were graphed with Kaplan Meier plots and were compared with log-rank test. Cox proportional hazard regressions were used to determine the unadjusted and the adjusted associations of PCS and MCS quintiles with each outcome. Hazard ratios (HR) and associated 95% confidence intervals (CI) were the measures of associations.

Interaction term between PCS and MCS for each outcome was tested prior to model building. A pre-specified set of variables, based on literature review, was selected for model adjustment [1,8,10,11,22]. Variables included participants’ demographic (age at screening, ethnicity, education, marital status), lifestyle (BMI, physical activity, fruit and vegetable consumption, smoking and drinking habits at study entry), and health characteristics [diabetes, hypertension, COPD (asthma or emphysema), cancer (except non-melanoma skin cancer), arthritis, medication use for high cholesterol, and systolic and diastolic blood pressure at baseline].

For each outcome, analyses were adjusted for confounding variables. Models examining the association of PCS and MCS on outcomes were sequentially adjusted for age at screening, then demographic, lifestyle and health characteristics, and finally for MCS (if PCS was the exposure) or PCS (if MCS was the exposure). A sensitivity analysis, where outcomes that occurred in the first year of follow-up were excluded, was also conducted. In addition, adjusted models were used to assess all eight subscales of SF-36 with the outcomes. The aforementioned covariates were used for the adjustment.

Participants with CVD at baseline were excluded from the incident CVD and CVD-specific death models. To examine the effect of CVD at baseline, the all-cause death model was run with and without participants who had CVD at baseline. The all-cause death model presented in the tables and graphs included those with CVD at baseline. All tests were two-sided and analyses were conducted in SAS version 9.2 (Cary, NC).

## Results

### Sample characteristics

Women in the analytic sample (n=20,308) had a mean age of 62.8 years [standard deviation (SD): 6.9] and a mean BMI of 28.9 kg/m^2^ (SD: 5.9); 37.2% were college graduates, 63.1% were married, and 38.6% were employed. Participants self-identified their race/ethnicity as: White = 82.3%, African-American = 9.9%, Hispanic/Latino = 3.5%, Asian/Pacific Islander = 2.3%, and American Indian/Alaskan Native = 0.4%.

### Sample characteristics by PCS and MCS quintiles (Q)

Participants’ PCS and MCS score were strongly associated with demographic, life-style, and disease-related variables but the distributions across the quintiles differed (Tables 1 &2). For example, whereas the mean age decreased incrementally across the quintiles of PCS, it increased across the quintiles of MCS. In the lowest PCS quintile (reference highest quintile), the proportion of participants with obesity was greater by 3 times, with diabetes by roughly 6 times, and with HTN, CVD, COPD, or arthritis by nearly 3 times. On the other hand, the proportions of participants with these conditions were similar between the lowest and the highest MCS quintiles (Tables 1 &2). PCS and MCS quintiles were significantly correlated with one another (spearman correlation: -0.12, p-value <0.0001).

**Table 1:** Association of physical component summary (PCS) quintile with demographics, life-style and health-related factors (n =20,308)

	**Physical component summary quintile**					
	**Q1 0–40.1 (n = 4062)**	**Q2 40.2 - 48.3 (n = 4061)**	**Q3 48.4 - 52.6 (n = 4058)**	**Q4 52.7 - 55.9 (n = 4066)**	**Q5 56–75 (n = 4061)**	
**Baseline variable**	**Mean (sd) or percent**	**Mean (sd) or percent**	**Mean (sd) or percent**	**Mean (sd) or percent**	**Mean (sd) or percent**	**p-value**
Age^a^	64.4 (7.0)	63.7 (7.0)	62.9 (6.8)	62.3(6.8)	60.9(6.5)	<0.0001
Age (category)						<0.0001
50–59	26.4	29.2	32.6	36.1	44.2	
60–69	47.9	48.3	48.7	47.7	44.2	
70-80	25.7	22.5	18.7	16.2	11.6	
Ethnicity						<0.0001
White	79.3	81.3	82.7	83.9	84.9	
African American	13.7	11.1	9.6	8.3	6.7	
Hispanic/Latino	3.5	3.2	3.2	3.1	3.4	
Asian/PI	1.4	2.6	2.7	3.0	3.2	
AI/AN	0.5	0.4	0.3	0.4	0.3	
Other	1.3	1.3	1.3	1.1	1.3	
Marital Status						<0.0001
Never married	4.1	3.9	4.2	4.0	4.0	
Divorced/widowed	37.8	34.2	30.6	29.7	31.8	
Currently married	58.1	61.8	65.2	66.3	64.2	
Education						<0.0001
< high-school	7.8	5.7	4.1	3.5	2.9	
High-school	20.6	20.4	19.3	16.9	15.8	
Some-college	43.8	40.0	39.7	38.0	35.8	
College-graduate	27.7	33.9	36.8	41.6	45.4	
BMI( kg/m^2^)^a^	31.8(7.0)	29.7(5.9)	28.6(5.4)	27.6(5.2)	26.6(4.5)	<0.0001
BMI (category)						<0.0001
<25	16.4	22.7	27.0	34.4	40.2	
25–29.9	27.8	34.2	38.4	38.5	39.5	
≥ 30	55.8	43.1	34.6	27.1	20.3	
Physical activity (MET/wk)						<0.0001
< 90	36.4	26.7	22.7	17.0	16.2	
90–449	31.1	31.0	28.6	27.4	23.6	
450–600	17.7	21.5	22.9	23.5	21.9	
> 600	14.8	20.8	25.8	32.1	38.2	
FV						0.009
<3 servings/d	43.3	41.3	40.0	39.9	41.5	
3–5 servings/d	34*.*5	35.8	37.3	36.2	35.8	
5+ servings/d	22.2	22.9	22.7	23.8	22.6	
Pack-years^a^	12.1 (20.6)	10.3(18.4)	9.7(17.7)	8.5(15.7)	8.8(16.0)	<0.0001
Smoking status						0.10
Never Smoked	51.6	53.5	54.4	55.1	52.9	
Past Smoker	40.3	38.7	38.2	37.9	39.5	
Current Smoker	8.2	7.9	7.5	7.0	7.6	
Alcohol intake						<0.0001
Non drinker	12.1	11.9	11.7	9.0	8.1	
Past drinker	27.7	21.0	16.6	15.0	13.8	
Current drinker	60.2	67.1	71.7	76.0	78.1	
Diabetes = Yes	12.6	8.2	6.3	3.7	2.2	<0.0001
COPD = yes	16.3	11.8	8.7	7.1	5.4	<0.0001
Arthritis = yes	73.8	57.9	44.2	32.6	23.2	<0.0001
CVD = Yes	27.1	19.2	15.0	11.2	9.1	<0.0001
Ever Cancer = yes	6.2	4.7	3.9	3.6	3.6	<0.0001
HTN = yes	50.8	40.6	35.6	28.8	20.7	<0.0001
Systolic BP ^a^	131.3(17.5)	129.6(17.5)	128.8(17.7)	126.7(17.0)	123.9(16.6)	<0.0001
Diastolic BP ^a^	76.3(9.4)	75.9(9.1)	76.2(9.2)	75.8(8.9)	75.1(8.7)	<0.0001
MCS ^a^	54.1(9.3)	53.6(8.1)	53.9(7.2)	54.2(6.5)	50.5(9.5)	<0.0001

^a^ Data are presented as mean (SD); all other data are presented as percent per quintile.

PI = Pacific Islander, AI = American Indian, AN = Alaskan Native, BMI = body mass index, BP = blood pressure, COPD = Chronic obstructive pulmonary disease (asthma or emphysema), CVD = cardiovascular diseases, FV = fruits and vegetables, HTN = hypertension, MCS = Mental component summary, MET = Metabolic equivalent of task, Q = quintile, SD = standard deviation.

**Table 2:** Association of mental component summary (MCS) quintile with demographics, life-style, and health-related factors (n =20,308)

	**Mental component summary quintile**					
	**Q1 0 – 48.0 (n = 4061)**	**Q2 48.1 – 54.0 (n = 4063)**	**Q3 54.1 – 57.0 (n = 4061)**	**Q4 57.1 – 59.3 (n = 4065)**	**Q5 59.4 - 75 (n = 4058)**	
**Baseline variable**	**Mean (sd) or percent**	**Mean (sd) or percent**	**Mean (sd) or percent**	**Mean (sd) or percent**	**Mean (sd) or percent**	**p-value**
Age ^a^	61.8(7.2)	62.2(6.8)	62.5(6.8)	63.0(6.7)	64.8(6.8)	<0.0001
Age (category)						<0.0001
50–59	41.2	37.0	35.7	31.5	22.9	
60–69	41.8	46.8	47.1	50.1	50.9	
70-80	16.9	16.1	17.1	18.3	26.2	
Ethnicity						<0.0001
White	78.6	81.2	84.4	84.6	83.3	
African American	12.7	10.4	8.4	8.4	9.5	
Hispanic/Latino	4.1	3.9	3.0	2.6	2.8	
Asian/PI	2.3	2.5	2.7	2.8	2.6	
AI/AN	0.6	0.3	0.3	0.4	0.3	
Other	1.5	1.5	0.9	1.0	1.3	
Marital Status						
Never married	4.3	4.2	4.2	3.5	4.0	<0.0001
Divorced/Widowed	38.7	33.3	29.3	29.7	33.3	
Currently married	57.0	62.5	66.7	66.8	62.7	
Education						
Less than high-school	6.7	5.2	4.4	3.0	4.7	<0.0001
High-school	20.5	19.1	18.1	17.3	18.0	
Some-college	39.7	39.2	38.2	38.5	41.6	
College-graduate	33.1	36.5	39.2	41.0	35.6	
BMI( kg/m^2^) ^a^	29.3(6.2)	28.8(5.9)	28.3(5.7)	28.2(5.5)	29.7(6.3)	<0.0001
BMI (category)						
<25	25.0	28.9	31.2	31.3	24.2	<0.0001
25–29.9	35.6	35.7	36.7	36.3	34.1	
≥ 30	39.4	35.4	32.1	32.4	41.7	
Physical activity (MET/wk)						
< 90	30.2	25.1	20.4	19.3	24.2	<0.0001
90–449	29.7	29.7	27.9	27.4	26.9	
450–600	18.8	21.0	23.1	22.3	22.3	
> 600	21.3	24.2	28.6	31.0	26.6	
FV						0.009
<3 servings/d	48.5	43.6	40.0	36.3	37.7	
3–5 servings/d	31.6	35.8	36.5	38.8	37.0	
5+ servings/d	19.9	20.6	23.5	24.9	25.3	
Pack-years ^a^	11.1 (19.2)	10.0(17.6)	9.4(17.4)	9.2(16.8)	9.6(17.8)	<0.0001
Smoking status						
Never Smoked	51.4	52.1	53.7	53.8	56.4	<0.0001
Past Smoker	38.0	39.0	39.6	40.1	37.9	
Current Smoker	10.6	8.9	6.7	6.2	5.7	
Alcohol intake						
Non drinker	9.8	9.5	10.1	10.9	12.5	<0.0001
Past drinker	22.0	20.1	17.4	15.6	19.0	
Current drinker	68.2	70.4	72.5	73.6	68.5	
Diabetes = Yes	8.0	6.9	6.1	4.5	7.0	<0.0001
COPD = yes	12.3	10.2	8.2	8.4	10.2	<0.0001
Arthritis = yes	50.6	44.2	41.1	41.6	54.3	<0.0001
CVD = Yes	18.7	16.8	15.5	13.8	16.8	<0.0001
Ever Cancer = yes	4.7	5.4	4.1	4.0	3.8	<0.0001
HTN = yes	37.5	36.0	33.4	32.6	36.9	<0.0001
Systolic BP ^a^	127.6(17.5)	127.7(17.6)	127.5(17.4)	127.9(17.2)	129.6(17.3)	<0.0001
Diastolic BP ^a^	76.1(9.1)	75.8(9.3)	75.8(9.2)	75.8(8.9)	75.8(9.1)	0.69
MCS ^a^	48.5(10.8)	48.8(8.9)	49.4(8.4)	49.5(8.3)	43.7(10.4)	<0.0001

^a^ Data are presented as mean (SD); all other data are presented as percent per quintile.

PI = Pacific Islander, AI = American Indian, AN = Alaskan Native, BMI = body mass index, BP = blood pressure, COPD = Chronic obstructive pulmonary disease (asthma or emphysema), CVD = cardiovascular diseases, FV = fruits and vegetables, HTN = hypertension, MET = Metabolic equivalent of task, PCS = Physical component summary, Q = quintile, SD = standard deviation.

### Unadjusted associations of PCS and MCS quintiles with CVD incidence, CVD death, and all-cause death

**Figure 2:**
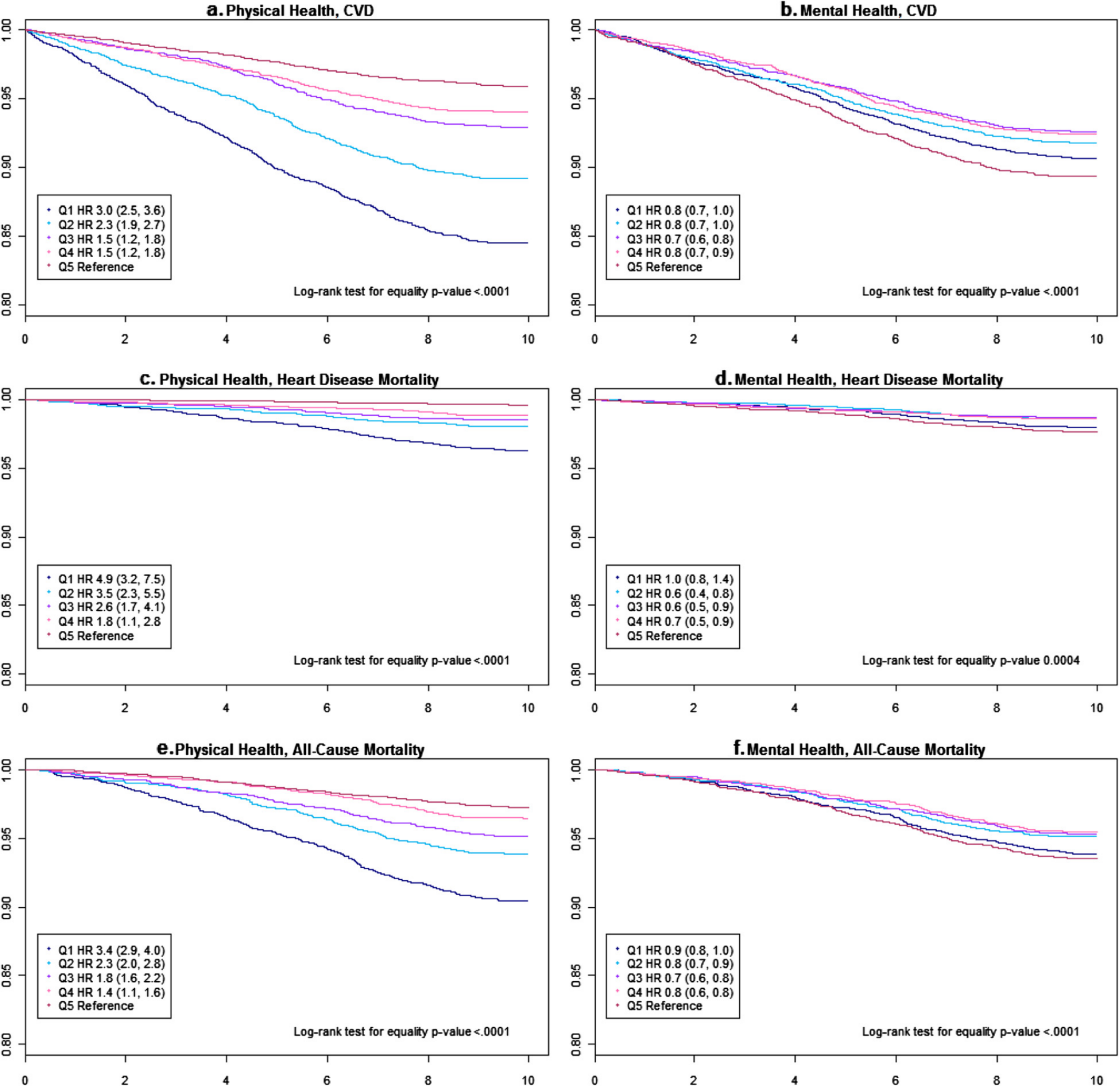
**Unadjusted plots for study outcomes.** (**a-f**) Kaplan Meier survival plots of cardiovascular (CVD) incidence, CVD-specific and all-cause death (n = 20,308). The hazard ratios and the associated 95% CI are the unadjusted estimates for Physical Component Summary (PCS) and Mental Component Summary (MCS) quintiles (Q).

Kaplan-Meier plots of time to CVD incidence, CVD-specific death, or all-cause death were significant for both PCS and MCS. Unadjusted Cox model showed that risk estimates (for all 3 outcomes) increased in the comparison PCS quintiles and they decreased in the comparison MCS quintiles (compared to respective reference quintile, Figure 2). Interaction term between PCS and MCS was not significant for any of the outcomes (p values >0.25).

### Adjusted associations of PCS and MCS quintiles with CVD incidence, CVD death, and all-cause death

Age-adjustment substantially reduced the risk estimates of PCS quintiles; however, the HR remained significant for each comparison quintile (Q1-Q4) for CVD incidence and CVD death, and for the lowest 3 quintiles (Q1-Q3) for all cause death. Adjustment for the remaining covariates, except MCS, attenuated the risks further, and for CVD incidence the HR of Q4 and Q3 quintile lost statistical significance (Data not shown). Additional adjustment for MCS scores hardly made any difference to the risk estimates (Table 3). For all-cause death, the estimates in the fully adjusted model did not change when participants with CVD at baseline were excluded (Data not shown).

**Table 3:** Adjusted association of cardiovascular (CVD) incidence, CVD-specific, and all-cause death (1993–2005; n = 20,308)

**SF 36 Physical summary component quintile**						
	**Q1: 0–40.1 (n = 4062)**	**Q2: 40.2 - 48.3 (n = 4061)**	**Q3: 48.4 - 52.6 (n = 4058)**	**Q4: 52.7 - 55.9 (n = 4066)**	**Q5: 56–75 (n = 4061)**	
Events/N	HR (95% CI)	HR (95% CI)	HR (95% CI)	HR (95% CI)	Ref	p-value
CVD incidence ^a^ 1075/16994	1.80 (1.43, 2.27)	1.57(1.25, 1.96)	1.09(0.86, 1.37)	1.25(0.99, 1.56)	1.00	<0.0001
CVD death ^a^ 204/16994	4.66 (2.31, 9.43)	3.53 (1.75, 7.11)	3.05 (1.50, 6.19)	2.97 (1.45, 6.07)	1.00	0.0007
All-cause death ^a b^ 1043/20308	2.14 (1.68, 2.73)	1.63 (1.29, 2.07)	1.44 (1.13, 1.84)	1.18 (0.92, 1.53)	1.00	<0.0001
**SF 36 Mental summary component quintile**						
	**Q1: 0 – 48.0 (n = 4061)**	**Q2: 48.1 – 54.0 (n = 4063)**	**Q3: 54.1 – 57.0 (n = 4061)**	**Q4: 57.1 – 59.3 (n = 4065)**	**Q5: 59.4 - 75 (n = 4058)**	
Events/N	HR (95% CI)	HR (95% CI)	HR (95% CI)	HR (95% CI)	Ref	p-value
CVD incidence ^a^ 1075/16994	0.96 (0.80, 1.17)	0.93 (0.77, 1.16)	0.82 (0.68, 0.99)	0.99 (0.82, 1.20)	1.00	0.29
CVD death ^a^ 204/16994	1.33 (0.90, 1.95)	0.63 (0.39, 1.01)	0.76 (0.48, 1.19)	0.87 (0.56, 1.35)	1.00	0.02
All-cause death ^a b^ 1043/20308	1.10 (0.91, 1.32)	0.93 (0.77, 1.13)	0.95 (0.79, 1.16)	0.96 (0.79, 1.17)	1.00	0.46

^a^ Models were adjusted for age, ethnicity, education, marital status, body mass index, fruits and vegetables (servings/day), physical activity (MET/wk), smoking history (pack-years), alcohol consumption status, systolic and diastolic blood pressure (mmHg), medication for hypertension or cholesterol, and diabetes, Chronic obstructive pulmonary disease (asthma and emphysema), arthritis, cancer at baseline, mental component summary (when physical component summary was the exposure), and physical component summary (when mental component summary was the exposure).

^b^ Also adjusted for CVD at baseline in addition to the variables mentioned above.

The risk estimates of the comparison MCS score quintiles changed in successive adjustment steps: first with age adjustment, then with all other selected covariates (except PCS), and finally with PCS scores. The HRs of final model for each outcome had a very modest U-shaped pattern of associations, although the comparison quintiles were not significant, except the 3rd quintile in relation to CVD incidence (Table 3).

### Sensitivity analysis (PH quintiles & the selected outcomes)

When the events that occurred in the first year of the trial were excluded, participants’ PCS scores remained significantly associated with CVD incidence, CVD-specific death, and all-cause death. A comparison of HRs between the adjusted models, with or without the exclusions of events, indicated little change in the estimate and no change in the significance (data not shown).

### Adjusted associations of SF-36 subscales with CVD incidence, CVD death, and all-cause death

Fully adjusted models showed that among the eight subscales of SF-36, physical functioning was significantly associated with all outcomes; each 10-point increase in the physical functioning score was associated with 7% reduction in CVD incidence, 13% reduction in CVD specific death, and 8% reduction in all-cause death. Additionally, general health perception was significantly associated with all-cause death. The other subscales were not significantly associated with any outcome (Additional file 2: Table S2).

## Discussion

This report shows that physical functional health, as derived from the SF-36 HRQOL, is a significant independent predictor of CVD incidence in older women. Specifically, women who were in the bottom 2 quintiles (Q1, Q2) were at 80% and 60% higher risk of developing CVD, respectively, than those in the top quintile (Q5). The significant risk of CVD associated with low PCS scores exceeds the risk of other lifestyle related CVD risk factors such as obesity, physical inactivity, smoking, and poor dietary habit associated with low PCS. Further this study validates the EPIC-Norfolk and NHS findings by showing that a low PCS score is a strong predictor of death, either from CVD or from all-causes [1,14].

A couple of hypothesis such as ‘disease severity’ and ‘subclinical disease’ have been proposed to explain the associations of PCS with mortality (i.e. individuals with a low PCS score are either overtly diseased or have disease that are not clinically manifested yet). These hypotheses cannot be rejected with the present analysis; however, they are unlikely to explain fully the findings of this study. The strong association of low PCS scores on CVD incidence and death, despite exclusion of events in the first year (sensitivity analysis) and inclusion of indicators of poor health in the model (e.g. diagnosis with diabetes, hypertension, COPD, arthritis, or cancer, use of medication for hypertension or high cholesterol), argues against the ‘disease severity’ hypothesis. Similarly, long follow-up data (mean 9.2 years) and inclusion of CVD related marker (e.g. blood pressure) in the model argues against the ‘subclinical disease’ hypothesis.

A reflection on the questions that constitute the PCS scale may provide insight on why PCS scores strongly predict CVD health and death. SF-36 items that composes the PCS scale ask participants to assess their general health while others inquire about bodily pain, the level of ease with which they can perform certain types of physical activities of daily living, and the activities that they recently had to cut back due to reduced physical function. The questions may capture several psychological constructs indirectly in addition to measuring perceived physical health directly. For example, high PCS scores may reflect higher levels of optimism while low scores may be indicative of higher levels of hopelessness and negative attitudes. In fact, these psychological constructs have been associated with CVD incidence and CVD specific mortality [23-25].

In this study, the MCS score, which was marginally associated with study outcomes when considered alone, showed no significant relationship in adjusted models. Most previous studies have similarly reported either a null or weak association between the mental domain of HRQOL and survival [8,26,27]. A possible explanation is that SF-36 is better at assessing mental health well-being and not designed to detect severe mental disorders, hence the lack of an association.

Beyond the summary scales (PCS, MCS), this study showed that physical functioning and general health subscale were two most important of the eight subscales of SF-36, in that they were significantly associated with the outcomes. Although these findings are similar to what have been reported in the literature [6,28], they should be taken with caution, as the subscales are highly correlated with one another, and hence their effects are not independent.

The implications of this study’s findings are potentially important to clinicians and researchers. First, PCS should be tested as a screening tool in the healthy adult population to identify those who are at-risk of CVD compared to the existing practices (e.g. blood pressure, serum lipid). Second, studies should explore whether an improvement (or decline) in PCS score over time may also be associated with a decreased (or increased) likelihood of CVD incidence. There is already evidence to show that improvements in PCS are associated with better survival, and that lifestyle interventions can be effective in reducing the rate of decline in PCS among older adults [14,22,29].

This study has several strengths, including a large sample, long follow-up, the use of a validated quality of life and physical activity scale, objective height, weight, and blood pressure measures, and detailed information on demographics, lifestyle factors, and health conditions, thereby enabling control for potential confounders. A further strength is the high quality comprehensive ascertainment of outcomes, including centrally adjudicated CVD and other health outcomes.

Despite adjustment for chronic conditions at baseline, there may be residual confounding in the analytical models and the effect of unmeasured confounding factors cannot be ruled out, both of which are potential limitations and may partly explain the results. Further, participants’ health conditions at baseline were self-reported.

## Conclusion

Perceived physical health was a strong predictor of incident CVD (fatal and non-fatal), CVD-specific death, and all-cause death in community dwelling postmenopausal women. Women in the lowest quintile of perceived physical health had substantially higher risk for incident CVD compared to women in the highest quintile. They also had higher risk of CVD specific and all-cause death. Future studies should test the value of PCS as a potential screening tool in older women to identify those who are at high risk for CVD development.

## Competing interest

There was no funding for this study. No support from any organization for the submitted work; no financial disclosures were reported by the authors of this paper.

## Authors’ contributions

All co-authors have contributed to this work to fulfill authorship criteria. The original idea belongs to NS and all co-authors worked on the protocol. NS and JK performed statistical analyses under the supervision of MD and MS. All co-authors drafted the final paper and all have approved the final version.

## Pre-publication history

The pre-publication history for this paper can be accessed here:

http://www.biomedcentral.com/1471-2458/13/468/prepub

## Supplementary Material

Additional file 1: Table S1 Names of the institutional review boards that approved the Women’s Health Initiative Study. Click here for file

Additional file 2: Table S2 Adjusted association of SF-36 subscales with cardiovascular (CVD) incidence, CVD-specific, and all-cause death (1993-2005). Click here for file
