# Supplementary material for: Self-perceived physical health predicts cardiovascular disease incidence and death among postmenopausal women
Source: BMC Public Health. 2013 May 14;13:468. doi: 10.1186/1471-2458-13-468 (PMC3706392; doi:10.1186/1471-2458-13-468)
Supplement: Additional file 1: Table S1 — Names of the institutional review boards that approved the Women’s Health Initiative Study. [file 1471-2458-13-468-S1.docx]

Supplemental Table 1. Names of the institutional review boards that approved the Women’s Health Initiative Study

| **Clinic #** | **Institution** | **Field Center** | **Protocol/ID #** |
| --- | --- | --- | --- |
| 12 | U of Alabama at Birmingham | Birmingham | F920828009 |
| 65 | U of Nevada | Nevada | B04/05-001 |
| 56 | U of Wisconsin | Madison | H-2004-0363 |
| 67 | U of TX Health Sci Ctr | San Antonio | 045-7000-050 |
| 64 | U of Miami | Miami | 20051390 |
| 26 | U of Med & Dentistry of NJ | Newark | 0120040296 |
| 60 | Rush-Presby-St Luke's Med Ctr | Chicago Rush | 94121901 |
| 29 | U of AZ Health Sciences Ctr | Tucson/Ph | 93-129 |
| 22 | U of CA, San Diego | La Jolla | 6136 |
| 45 | U of Hawaii | Honolulu | 9760 |
| 57 | State U of NY at Stony Brook | Stony Brook | 20065617 |
| 68 | U of CA, Los Angeles | Los Angeles | 04-08-062-04 |
| 43 | Medical College of Wisconsin | Milwaukee | 067-94 |
| 61 | U of Cincinnati Medical Ctr | Cincinnati | 93-08-24-04 EE |
| 53 | Kaiser Fndtn Research Inst | Oakland | CN-94RHIAT-02-H |
| 16 | Northwestern U | Chicago | 0499-006 |
| 47 | Baylor College of Medicine | Houston | H-11609 |
| 72 | U of Med & Dentistry of NJ | N Brunswick | 0219961844 |
| 19 | Emory U Sch of Medicine | Atlanta | 022-2004 |
| 25 | U of Minnesota | Minneapolis | 9308M07098 |
| 49 | A. Einstein College of Med | New York | 2004-151 |
| 23 | Memorial Hospital of Rhode Is | Pawtucket | 92-30A |
| 48 | U of Massachusetts | Worcester | H-11417 |
| 24 | U of Tenn Memphis | Memphis | 4554 |
| 30 | U of CA, Davis | Davis | 200210270-14 |
| 66 | Kaiser Fndtn Research Inst | Portland | 00000405 |
| 15 | State U of NY at Buffalo | Buffalo | SPM0290393A |
| 55 | Harbor-U of CA | Torrance | 07714-01 |
| 62 | Wayne State U | Detroit | 0409000121 |
| 46 | U of Florida | Gainesville | 376-2004 |
| 42 | Stanford U | Stanford | 95415 |
| 44 | George Washington U | GWU | 099304 |
| 13 | Bowman Gray Sch of Med | Winston Salem | BG00-316 |
| 51 | Medlantic Research Inst | MedStar | 1994-037 |
| 18 | FHCRC | Seattle | 3493 |
| 63 | U of CA, Irvine | Irvine | 1993-335 |
| 58 | U of NC at Chapel Hill | Chapel Hill | 04-EPID-486 |
| 21 | U of Iowa | Iowa City | 199309487 |
| 28 | U of Pittsburgh | Pittsburgh | 0404092 |
| 50 | Ohio State U | Columbus | 1994H0412 |
| 14 | Brigham & Women's Hospital | Boston | 1999-P-001544 |
|  | FHCRC - Coordinating Ctr | CCC | 3467 |
