# Supplementary material for: Self-perceived physical health predicts cardiovascular disease incidence and death among postmenopausal women
Source: BMC Public Health. 2013 May 14;13:468. doi: 10.1186/1471-2458-13-468 (PMC3706392; doi:10.1186/1471-2458-13-468)
Supplement: Additional file 2: Table S2 — Adjusted association of SF-36 subscales with cardiovascular (CVD) incidence, CVD-specific, and all-cause death (1993-2005). [file 1471-2458-13-468-S2.docx]

Supplementary Table 2: Adjusted association of SF-36 subscales with cardiovascular (CVD) incidence, CVD-specific, and all-cause death (1993-2005)

|  |  | **CVD Incidence** ^a^ | | **CVD-specific Death** ^a^ | | **All-cause Death** ^a b^ | | |
| --- | --- | --- | --- | --- | --- | --- | --- | --- |
| *Subscale* | *Mean ± SD* | *HR (95% CI)* ^c^ | *p-value* | *HR (95% CI)* ^c^ | *p-value* | *HR (95% CI)* ^c^ | *p-value* |  |
| SF | 90.2 ± 17.4 | 1.01 (0.97, 1.05) | 0.5954 | 1.00 (0.92, 1.08) | 0.9071 | 1.00 (0.97, 1.03) | 0.9669 |  |
| PF | 80.7 ± 19.7 | 0.93 (0.90, 0.97) | <.0001 | 0.87 (0.81, 0.93) | <.0001 | 0.92 (0.89, 0.94) | <.0001 |  |
| RP | 74.4 ± 35.0 | 1.00 (0.98, 1.02) | 0.9215 | 1.00 (0.96, 1.04) | 0.9921 | 0.99 (0.97, 1.01) | 0.3114 |  |
| BP | 74.5 ± 22.9 | 0.99 (0.96, 1.03) | 0.6232 | 1.05 (0.98, 1.12) | 0.1605 | 1.00 (0.98, 1.03) | 0.8706 |  |
| GH | 74.2 ± 17.2 | 0.96 (0.92, 1.00) | 0.0525 | 0.95 (0.87, 1.04) | 0.2483 | 0.94 (0.91, 0.97) | 0.0004 |  |
| VT | 62.9 ± 19.0 | 0.98 (0.94, 1.03) | 0.4296 | 0.97 (0.89, 1.05) | 0.4535 | 0.98 (0.95, 1.02) | 0.3538 |  |
| MH | 79.1 ± 14.1 | 1.05 (1.00, 1.11) | 0.0746 | 1.08 (0.97, 1.20) | 0.1498 | 1.04 (1.00, 1.09) | 0.0805 |  |
| RE | 84.5 ± 29.0 | 1.00 (0.98, 1.02) | 0.8988 | 0.96 (0.92, 1.01) | 0.0953 | 1.00 (0.99, 1.02) | 0.6537 |  |

Abbreviations: SF: social functioning, PF: physical functioning, RP: role limitations due to physical health, BP: bodily pain, GH: general health perceptions, VT: vitality, MH: mental health, RE: role limitations due to emotional problems.

^a^ Models were adjusted for age, ethnicity, education, marital status, body mass index, fruits and vegetables (servings/day), physical activity (MET/wk), smoking history (pack-years), alcohol consumption status, systolic and diastolic blood pressure (mmHg), medication for hypertension or cholesterol, and diabetes, Chronic obstructive pulmonary disease (asthma and emphysema), arthritis, and cancer at baseline.

^b^ Also adjusted for CVD at baseline in addition to the variables mentioned above.

^c^ Hazard ratio (10-unit change)

All cause death (n =20,308), CVD incidence and CVD specific death (n = 16,994)
